# Development of a platform for broadband, spectra-fitted, tissue optical phantoms

**DOI:** 10.1117/1.JBO.28.2.025001

**Published:** 2023-02-20

**Authors:** Alec B. Walter, E. Duco Jansen

**Affiliations:** a Vanderbilt University, Department of Biomedical Engineering, Nashville, Tennessee, United States; b Vanderbilt University, Biophotonics Center, Nashville, Tennessee, United States; c Vanderbilt University Medical Center, Department of Neurosurgery, Nashville, Tennessee, United States

**Keywords:** optical properties, reflectance, absorption, scattering, standards, spectroscopy

## Abstract

**Significance:**

Current methods of producing optical phantoms are incapable of accurately capturing the wavelength-dependent properties of tissue critical for many optical modalities.

**Aim:**

We aim to introduce a method of producing solid, inorganic phantoms whose wavelength-dependent optical properties can be matched to those of tissue over the wavelength range of 370 to 950 nm.

**Approach:**

The concentration-dependent optical properties of 20 pigments were characterized and used to determine combinations that result in optimal fits compared to the target properties over the full spectrum. Phantoms matching the optical properties of muscle and nerve, the diffuse reflectance of pale and melanistic skin, and the chromophore concentrations of a computational skin model with varying oxygen saturation (${\mathrm{StO}}_{2}$) were made with this method.

**Results:**

Both optical property phantoms were found to accurately mimic their respective tissues’ absorption and scattering properties across the entire spectrum. The diffuse reflectance phantoms were able to closely approximate skin reflectance regardless of skin type. All three computational skin phantoms were found to have emulated chromophore concentrations close to the model, with an average percent error for the ${\mathrm{StO}}_{2}$ of 4.31%.

**Conclusions:**

This multipigment phantom platform represents a powerful tool for creating spectrally accurate tissue phantoms, which should increase the availability of standards for many optical techniques.

## 1 Introduction

The development and validation of optical instrumentation for medical diagnosis, surgical guidance, and light-based therapeutics often require the use of well-characterized phantoms as standardized samples. Their uses include determining detection limits, assessing spectral and spatial resolution, validating postprocessing algorithms, and performing quality control over time and between different systems, among others.^1^^,^^2^ Although an ideal phantom would be one that could be used for all applications and modalities, this would at least require a solid phantom that has tunable optical properties with tissue-like wavelength dependence over a broad spectral range, while being stable over time and throughout multiple uses, something that current methods cannot achieve.

Phantoms with wavelength-dependent optical properties similar to tissue are usually achieved by directly using biological chromophores, such as whole blood or hemoglobin, to provide absorption and lipid emulsions, such as Intralipid, to provide scattering.^3^^–^^7^ Though simple aqueous solutions can be used, these additions can also be suspended in a biologically compatible gelatin- or agar-based hydrogel to produce solid, moldable phantoms.^6^^,^^8^^–^^10^ Although this can result in spectrally accurate phantoms for a wide range of tissue types, the organic nature of both the chromophores and matrix materials inherently limits the shelf-life of these phantoms to somewhere between a few hours and a couple weeks, depending on environmental conditions.^1^^,^^5^^,^^6^^,^^9^ As such, they are typically made for a single task and discarded after use, preventing them from serving as effective standards.

To make phantoms whose optical properties are stable over long periods of time, transparent, inorganic matrix materials, such as silicone or resin, are used as they are resistant to environmental effects and can result in effective shelf-lives of at least several years. However, this stability comes with the trade-off of these materials being incompatible with the inclusion of biological chromophores, preventing them from easily matching the spectral optical properties of tissue.^1^ Instead, the optical properties of these phantoms are typically controlled through the addition of a metal oxide powder, usually either titanium dioxide (${\mathrm{TiO}}_{2}$) or aluminum oxide (${\mathrm{Al}}_{2}O_{3}$), or polystyrene microspheres to provide scattering and a carbon-based black pigment, usually in the form of either India ink or nigrosin, to provide flat absorption across the spectrum.^8^^,^^9^^,^^11^^–^^18^ By varying the concentrations of these additions, nearly any combination of absorption and scattering coefficients can be achieved at a specified wavelength.

Although this approach works well when only a single wavelength is of interest, it is incapable of accurately capturing the wavelength-dependent scattering and absorption properties of tissue. Having such a dependence can be important for many optical modalities, such as fluorescence imaging, as both the properties at the excitation and emission wavelengths impact the collected emitted signal, chromophore extraction techniques, including pulse oximetry and near-infrared spectroscopy, which rely on the absorption at two or more distinct wavelength bands to approximate the concentrations of tissue chromophores and other spectroscopic techniques. Many groups have attempted to address this need through the addition of more specific absorbers with the inclusion of red ink being a relatively popular choice as it provides higher absorption in the green and blue regions of the spectrum while maintaining a low absorption in the red and NIR.^19^ Cosmetic powders matched to different skin-tones have also been used to provide similar spectral absorption differentials, though are less commonly used due to their comparatively high scattering.^20^ Different molecular dyes with relatively narrow absorption peaks have also been used in order to independently tune two separate wavelengths to the desired properties.^15^^,^^21^ However, to our knowledge, there has yet to be a phantom design, which is capable of mimicking the wavelength-dependence of an arbitrary tissue across the VIS-NIR spectrum.

To address this, we have developed a platform capable of producing solid, stable tissue phantoms whose absorption and scattering properties can be spectrally matched the desired tissue properties over a range 370 to 950 nm. This was accomplished by first characterizing 20 commercially available pigments which provide either scattering, baseline absorption, or absorption peaks throughout blue, green, and red wavelengths. By feeding the concentration-dependent properties of the pigments into a nonnegative, least squares optimization, the combination of pigments which results in a phantom with wavelength-dependent optical properties highly similar to those of the target tissue can be determined. To assess the accuracy and scope of this method, phantoms were made matching the optical properties of human tissues for either the full spectrum or selected wavelength bands within the full spectrum. The efficacy of the phantom platform was further assessed by making more complicated phantom designs including diffuse reflectance matched phantoms and chromophore mimicking phantoms.

## 2 Materials and Methods

### 2.1 Phantom Component Selection

Optical tissue phantoms typically consist of three basic components: a matrix material, an absorbing agent, and a scattering agent. For the matrix material, we chose to use a two-part epoxy resin (JANCHUN Crystal Clear Epoxy Resin Kit) due to its relatively quick cure time and inexpensive material cost compared to other common matrix materials, such as PDMS. The epoxy resin consists of a base material (part A) and a hardener (part B), which are mixed together using a 1A:1B volume ratio or 1A:0.9B weight ratio. After mixing, the epoxy has a pot life of 30 to 40 min where it can still easily be manipulated and will fully cure after 24 h at room temperature. To mitigate the risk of any additives settling, the cure time can be reduced to ∼30  min by elevating the temperature to 70°C.

To better mimic the complex and varied absorption and scattering spectra that can be found within tissue, we used 20 different pigments as shown in Table 1. For these pigments, we chose to utilize artist-grade acrylic paints whenever possible as opposed to the traditionally used powdered components. This was due to the fact that in our initial testing, we found that the included surfactants and stabilizers in acrylic paint made homogenizing the phantom easier to achieve without the need for sonication while also helping to prevent the scatterers from settling to the bottom of the epoxy, issues typically found when making solid, inorganic phantoms.

**Table 1:** Absorbing and scattering pigments used in the multipigment phantom platform.

Pigment	Brand	Color name	Lightfastness
*Yellow*			
PY3	Liquitex: Basics acrylic	Cadmium yellow light hue	II
PY65	Winsor & Newton: Galeria acrylic	Cadmium yellow deep hue	I
PY74	Liquitex: Basics acrylic	Primary yellow	I
PY100	Wilton: Gel icing color	Lemon yellow	—
PY154	Golden: Fluid acrylics	Benzimidazolone yellow medium	I
PY175	Golden: Heavy body acrylics	Benzimidazolone yellow light	I
*Magenta*			
PR122	Golden: Heavy body acrylics	Quinacridone magenta	I
PR170	Winsor & Newton: Galeria acrylic	Crimson	I
PR202	Plaza Art: Artists’ acrylic	Quinacridone fuchsia	I
PV15	Plaza Art: Artists’ acrylic	Ultramarine violet	I
PV19	Golden: Heavy body acrylics	Quinacridone violet	I
PV23	Plaza Art: Artists’ acrylic	Dioxazine purple	II
*Miscellaneous*			
PG7	Golden: Heavy body acrylics	Phthalo green (blue shade)	I
PB15:3	Golden: Heavy body acrylics	Phthalo blue (green shade)	I
PO73	Golden: Heavy body acrylics	Pyrrole orange	I
*White*			
PW4	Golden: Heavy body acrylics	Zinc white	I
PW6	Plaza Art: Artists’ acrylic	Titanium white	I
${\mathrm{Al}}_{2}O_{3}$	The Rock Shed	${\mathrm{Al}}_{2}O_{3}$ polish (≤2 μm)	—
*Black*			
Black 2.0	Culture Hustle	Black 2.0	—
India ink	Speedball	Super black	—

Additionally, the pigments used in artist-grade acrylic paint typically have had their lightfastness, or relative degree in change due to light exposure, characterized using the ASTM standard test methods.^22^ This process exposes the material to either direct solar illumination or a Xenon arc lamp with a similar spectral curve for a total irradiance of $1260\;\mathrm{MJ}/m^{2}$ and assess the degree of change which occurs within the CIE 1976 color space. Depending on the magnitude of the change, a pigment can receive a rating of either I, excellent; II, good; or III, fair. Where applicable, the pigments chosen for this work were all rated as having a lightfastness of I, with only two being rated as having a rating of II (see Table 1). As such, any phantoms made using the chosen pigments are being expected to have high stability over time and usage.

To match the variable scattering slope of different tissue types, we chose to use three of the most common scattering agents used in solid phantoms, titanium dioxide (pigment white 6; PW6), zinc oxide (pigment white 4; PW4), and ${\mathrm{Al}}_{2}O_{3}$.^8^^,^^12^^–^^16^^,^^23^^,^^24^ Due to differences in refractive index and average particle size, these three white pigments have been shown to provide different scattering powers and slopes which can be combined to better match that of tissue.^16^^,^^23^

The typical absorption spectrum of tissue consists of three main regions that a broadband phantom would need to match using three different categories of absorbing pigments. First, there is a general baseline absorption arising from all present chromophores, which is observed in the red and near-infrared regions of the spectra. This can be matched using black pigments that have broadband absorption across the entire spectra. We chose to use two different black pigments for this work, India ink, which has become a standard absorber for optical phantoms, and Black 2.0, to have some variance in the shape of the baseline absorption.

On top of the baseline, tissue has two major absorption peaks due to presence of hemoglobin, one in the blue region and another in the green region of the spectrum. To match these two absorption peaks, we used two classes of colored pigments: yellow pigments which primarily absorb blue wavelengths and magenta pigments which primarily absorb green wavelengths. Although the absorption coefficients of most pigments have not before been reported, many studies have analyzed their diffuse reflectance spectra.^25^^–^^28^ Using reported reflectance spectra, we selected six yellow and six magenta pigments that have absorption peaks around 420 and 550 nm, respectively. Finally, to account to any absorption features not covered by these three categories, we included three miscellaneous pigments. Pigment orange 73 (PO73) was included as its absorption should fall between the yellow and magenta pigments, whereas pigment green 7 (PG7) and pigment blue 15:3 (PB15:3) were chosen as their reported reflectance spectra include significant red and NIR absorption which none of the other included pigments have.

### 2.2 Phantom Fabrication

Each epoxy phantom was made using the same general procedure with each component being measured by weight. To begin, the desired amounts of any of the yellow, magenta, and miscellaneous pigments are weighed out in a plastic weigh boat. Following this, water weighing ∼10% of the desired amount of base epoxy is added to thin and rehydrate the acrylic paints. The water is then mixed with the pigments using a glass stir rod until it becomes homogenous in color. After this, the weigh boat is placed back onto the analytical balance and the desired amount of part A of the epoxy is weighed out. The added part A is then mixed by hand with the thinned pigments until a smooth emulsion is achieved. To remove the added water, the mixture is placed in a vacuum chamber and brought to −750  mmHg until bubbles stop forming. This process is then repeated in a separate weigh boat to combine part B of the epoxy with the black and white pigments, making sure that the amount of water used is at least 30% more than the weight of the white pigments.

After removing the water, the part A mixture is poured into the part B one and mixed by hand until homogeneous. After mixing, an initial degassing is performed by placing the weigh boat into a vacuum chamber and bringing the pressure to −750  mmHg until the bubbles stop forming or 10 min pass. The resin tends to expand by a large degree during the initial portion of degassing, so it is important to use a large enough vessel. After the initial degassing, the epoxy mixture is poured into silicone molds of the desired shape and size. Silicone molds were chosen to provide smooth surfaces to the phantoms so that the effects of diffuse reflectance from the sample surface are prevented from confounding the optical property measurements. These molds are then placed back into the vacuum chamber for a final degassing using the same procedure previously described. After all the remnant air bubbles have been removed, the epoxy is left to cure in an oven set to 70°C for at least 30 min, after which it is solid enough to safely demold.

### 2.3 Optical Property Measurement

The optical properties of the epoxy materials were determined between 370 and 950 nm using the single integrating sphere, inverse adding–doubling (IAD) approach.^29^^,^^30^ IAD solves for the optical properties of a material using reflectance and transmittance measurements as well as the thickness and refractive index of the sample. It accomplishes this by iteratively solving the radiative transport equation using the adding–doubling method for different pairs of optical properties until a set of absorption and reduced scattering coefficients are found produce a reflectance and transmittance which match the measurements. The thickness of each sample was measured using digital calipers, whereas the refractive index of epoxy was estimated from the literature to be n=1.56.^16^ Additionally, as we did not collect any collimated transmission spectra, we assumed a fixed scattering anisotropy of g=0.8 for every sample based on the average refractive index and size distributions of the different scattering particles.^16^^,^^23^

The required diffuse reflectance and total transmittance measurements were taken using a dual-beam spectrophotometer (Cary 5000, Agilent) with an integrating sphere attachment (DRA-2500, Agilent). Sample reflection and transmission measurements were converted into reflectance and transmittance using a zero correction and baseline normalization. For reflectance, the baseline measurement is taken using a 99% diffuse reflectance standard at the sample port (USRS-99-020, LabSphere), whereas the zero measurement is taken by allowing the light into the sphere but leaving the sample reflectance port open, accounting for any light that may be incident on the sphere wall before the sample. For the transmittance, the zero measurement is taken by blocking the sample beam from entering the sphere, whereas the baseline is determined in the same manner as for reflectance. The diameter of the spot size at the sample was maintained at 2 mm for both measurements, which was always no more than 5% of the total area of the sample port to minimize the loss of laterally scattered light.

To obtain samples that were thin enough for the required transmission measurements, a 50×50  mm square silicone mold was used, adding a fraction of the total amount of epoxy such that the bottom of the mold was lightly coated. After curing, the thin samples were demolded and a ∼25×25  mm square cut from the center to mitigate any effects that a meniscus would have on the thickness. Although not accurate enough to produce a sample of a specified thickness, this process consistently resulted in thicknesses between 0.5 and 1.0 mm.

Although IAD has been shown to produce accurate determination of optical properties from integrating sphere measurements, a substantial crosstalk effect has been known to occur in the IAD calculated reduced scattering coefficient for wavelength ranges corresponding to significant absorption peak of the material. To address this, we utilized the two-stage IAD process as proposed by Xie et al. for all of our measurements.^31^^,^^32^ The first stage of this process is to run IAD as previously described. The second stage takes the resulting reduced scattering coefficient and fits it to a Mie–Rayleigh power law equation. IAD is then run a second time using the same reflectance and transmittance data with the scattering constrained to the values obtained from the fitted model. This process has been shown to not only correct the shape of the scattering slope but also correct the absorption peaks to their expected values.

### 2.4 Pigment Characterization

Before being used for tissue phantoms, the optical properties of each of the 20 pigments (Table 1) while suspended in epoxy resin were characterized. To accomplish this, the phantom fabrication protocol as previously described was followed to add individual pigments into 9.5 g of epoxy. This process was repeated three times for every pigment, using a different mass fraction, which we defined as the amount of pigment in milligrams divided by the total mass in grams, each time. As the relative absorption and scattering strengths of the pigments differed, different sets of mass fractions were used for each pigment. The absorption and reduced scattering coefficients of the individual pigment samples were determined using the approach previously described. The results were then normalized to their respective mass fractions and averaged together for each given pigment.

### 2.5 Tissue Optical Property Phantoms

Using the concentration-dependent optical properties of the different pigments, phantoms were made to match the optical properties of muscle and nerve from human cadavers, which our group previously reported.^33^ These two tissue types were chosen as they represent two possible extremes, with muscle having relatively high absorption and low scattering and nerve having the opposite.

To determine the amount of each pigment required to best match one of the sets of optical properties between 370 and 950 nm, a two-step, nonnegative least squares optimization was used. The first step of this process determined the concentration of yellow, magenta, black, and miscellaneous pigments, which would result in a phantom absorption spectrum that most closely matched the desired values using the following equation:

$$\arg\;\min_{x}\;\overset{950}{\underset{\lambda =370}{\sum }}{\left[\sqrt{w(\lambda )}\mu_{a}^{\text{tissue}}(\lambda )-\sqrt{w(\lambda )}C_{\mathrm{abs}}^{x}(\lambda )x\right]}^{2},$$ (1)

where x is the concentrations of the absorbing pigments, w(λ) is a weighting function, $C_{\mathrm{abs}}^{x}(\lambda )$ is the concentration-dependent absorption coefficients of those pigments, and $\mu_{a}^{\text{tissue}}(\lambda )$ is the target absorption coefficient spectrum. However, as the absorption of tissue can vary over at least two orders of magnitude across the visible and NIR regions of the spectrum, any solution to this process would prioritize fitting to the absorption peaks in the blue and green portions of the spectrum over the lower values in the red and NIR. To account for this, we applied a wavelength-dependent weight to the optimization based on the target absorption spectrum. The weight w(λ) at each wavelength was set as the maximum absorption coefficient divided by the absorption coefficient at that wavelength. This corrects for any differences in magnitude across the spectrum so that each wavelength is treated equally in the least-squares optimization.

The second step of the optimization process involved a second nonnegative least squares optimization for the white pigments and the reduced scattering coefficient:

$$\arg\;\min_{z}\;\overset{950}{\underset{\lambda =370}{\sum }}{\left[(\mu_{s}^{\prime \text{tissue}}(\lambda )-C_{\mathrm{scat}}^{x}(\lambda )x)-C_{\mathrm{scat}}^{z}(\lambda )z\right]}^{2},$$ (2)

where z is the concentrations of the white pigments, $C_{\mathrm{scat}}^{z}(\lambda )$ is their concentration-dependent reduced scattering coefficients, and $\mu_{s}^{\prime \text{tissue}}(\lambda )$ is the target reduced scattering coefficient spectrum. Although the weighting approach as used for the absorption coefficient was not required, the amount of scattering that the non-white pigments would contribute was an important consideration. As such, the predicted amount of scattering caused by the absorbing pigments, calculated from their concentrations x and concentration-dependent reduced scattering coefficients $C_{\mathrm{scat}}^{x}(\lambda )$ was subtracted from the target scattering beforehand.

Additionally, many phantoms may only require matching properties at a number of discreet wavelength bands instead of across the entire spectrum. To determine the pigment concentrations required for such a phantom, a single optimization step was used, in which sum of the squared residuals for each property is minimized using the following equation:

$$\arg\;\min_{q}\;\overset{950}{\underset{\lambda =370}{\sum }}[w_{a}(\lambda ){\left(\mu_{a}^{\text{tissue}}(\lambda )-C_{\mathrm{abs}}(\lambda )q\right)}^{2}+w_{s}(\lambda ){\left(\mu_{s}^{\prime \text{tissue}}(\lambda )-C_{\mathrm{scat}}(\lambda )q\right)}^{2}],\;$$ (3)

where q is the concentrations of all of the pigments, $C_{\mathrm{abs}}(\lambda )$ and $C_{\mathrm{scat}}(\lambda )$ are their concentration-dependent absorption and reduced scattering coefficients, and $\mu_{a}^{\text{tissue}}(\lambda )$ and $\mu_{s}^{\prime \text{tissue}}(\lambda )$ are the target optical properties. To select the bands of interest, strong weighting functions ($w_{a}(\lambda )$ and $w_{s}(\lambda )$) are applied to both sets of properties at the specified wavelengths while the weights outside those bands are set to 1. These weights can be manually adjusted to account for differences in magnitude between the bands and to preferentially fit to certain bands of interest over others. A phantom matching the optical properties of muscle at four bands was made. The chosen bands were 385 to 405 nm, 530 to 570 nm, 615 to 635 nm, and 825 to 865 nm as these regions spanned the full range of that the absorption coefficient of that muscle exhibits.

After determining the desired pigment concentrations using either of these methods (see Table S1 in the Supplementary Material), phantoms were made using the previously described fabrication process. Each of the phantoms were made using 38 g of epoxy to allow for lower concentrations of some of the pigments to fall above the 5 mg minimum of the analytical balance. To account for the orders of magnitude difference in absorption coefficient that these phantoms would have across the spectrum, each phantom was made into two thin samples of different thicknesses, one around 0.5 mm and the other between 1.5 and 2.5 mm. The optical properties of both were then determined. The first sample is thin enough to allow for measurable transmittance and reflectance at the blue absorption peak, whereas the second is thick enough for the low absorption in the NIR to have a significant effect on the measurements. From the results of these two samples, a composite absorption coefficient spectrum was made. The absorption from the thinner sample was used from 370 to 560 nm, and the thicker sample’s absorption was used from 620 to 950 nm. Between these two regions, the absorption coefficients were blended together using a sigmoid-weighted average.

### 2.6 Diffuse Reflectance Phantoms

In some cases, the optical properties of a tissue of interest may be unknown making it difficult to make a phantom to accurately calibrate or test the performance of an optical system. Additionally, many modalities rely on diffuse reflectance, which, while dependent on the absorption and scattering properties, is also dependent on the refractive index. With the refractive index of epoxy resin, and other common phantom matrix materials, being significantly higher than that of tissue, tissue phantoms that mimic optical properties are likely to not match the corresponding diffuse reflectance. This is especially relevant for many systems that are used for *in vivo* applications. To determine the feasibility of making optical phantoms directly matched to tissue diffuse reflectance, phantoms matching different skin types were made. From a NIST study, which assessed the diffuse reflectance of the inner forearm *in vivo*, two spectra were selected, one representing pale (NIST#74) and the other melanistic skin (NIST#44).^34^

To determine the pigment concentrations that would result in phantoms matched to the desired diffuse reflectance spectra R(λ), a nonlinear, nonnegative, least-squares model was used. This process utilizes a white Monte Carlo (wMC) to predict the phantom diffuse reflectance given the refractive index of the material (n=1.56) and the absorption and reduced scattering coefficients, calculated using the concentration-dependent properties of the pigments, $C_{\mathrm{abs}}(\lambda )$ and $C_{\mathrm{scat}}(\lambda )$.^35^^,^^36^ The optimization iterates the pigment concentrations q, which results in new wavelength-dependent optical properties, which are fed into the wMC to produce diffuse reflectance spectra which are then compared to the target spectrum in a least-squares manner:

$$\arg\;\min_{q}\;\overset{950}{\underset{\lambda =370}{\sum }}{\left[(R(\lambda )-\mathrm{wMC}(C_{\mathrm{abs}}(\lambda )q,C_{\mathrm{scat}}(\lambda )q,n)\right]}^{2}.$$ (4)

The diffuse reflectance phantoms were made using the determined pigment concentrations (see Table S2 in the Supplementary Material) and 38 g of epoxy. Using a 35×35  mm mold, this resulted in phantoms with an approximate thickness of 17 mm, which was found to be sufficiently optically thick for all wavelengths. The diffuse reflectance of the phantoms was measured using an integrating sphere and spectrophotometer in a similar manner as for the optical property measurements. However, to maximize the capture of deep, laterally scattered photons a larger, 25-mm-diameter aperture was used.

### 2.7 Oxygenation Saturation Phantoms

A common modality that uses the optical properties of tissue at multiple spectral bands is the determination of tissue hemoglobin oxygen saturation (${\mathrm{StO}}_{2}$). By measuring the absorption coefficient at two or more wavelengths, tissue chromophore concentrations, namely oxy- and deoxyhemoglobin, can be estimated by solving a series of equations.^37^^–^^40^ This reliance on multiple wavelengths has made using inorganic phantoms to accurately emulate tissue chromophores difficult. As such, we wanted to determine if the multipigment approach could create phantoms that accurately emulate physiological chromophore concentrations given different levels of ${\mathrm{StO}}_{2}$.

To accomplish this, a computational model of skin absorption was made using blood, melanin, water, and fat as the primary chromophores. The absorption coefficient spectrum of the theoretical skin was determined through a summation of the absorption coefficients of the pure chromophores, obtained from the literature, such that

$$\mu_{a}(\lambda )={\mathrm{StO}}_{2}BH\mu_{a}^{{\mathrm{HbO}}_{2}}(\lambda )+(1-{\mathrm{StO}}_{2})BH\mu_{a}^{\mathrm{Hb}}(\lambda )+W\mu_{a}^{\text{water}}(\lambda )+F\mu_{a}^{\text{fat}}(\lambda )+M\mu_{a}^{\mathrm{mel}}(\lambda ),$$ (5)

where H is the concentration of hemoglobin within blood, ${\mathrm{StO}}_{2}$ is the ratio of oxyhemoglobin to total hemoglobin, and B, W, F, and M are the volume fractions of blood, water, fat, and melanosomes, respectively, within the tissue.^41^^–^^45^

Using fixed volume fractions, tissue absorption spectra were produced between 470 and 950 nm using three different values for ${\mathrm{StO}}_{2}$: 30%, 50%, and 80%. The volume fractions for water and fat were set to 0.5 and 0.02, respectively, based on the values used in similar models.^38^^,^^39^ A low melanosome volume fraction of 0.0255% was selected so that the theoretical tissue would have an absorption spectrum representative of pale, minimally pigmented skin.^44^^–^^47^ The volume fraction of total blood was set to be 0.047 which, given a blood hemoglobin concentration of 2.3  mmol/L, corresponded to a total hemoglobin concentration of 110  μM, which falls within reported physiological ranges.^37^^,^^39^ Although not as important for ${\mathrm{StO}}_{2}$ measurements, the reduced scattering coefficient of the tissue model was set to be similar to be similar to that of skin. This was accomplished through a Mie-Rayleigh power law equation with values derived from the literature.^44^^,^^47^

Multipigment phantoms were made to match the theoretical absorption coefficients for each of the three ${\mathrm{StO}}_{2}$ values using 38 g of total epoxy. The required pigment concentrations (see Table S3 in the Supplementary Material) were determined using the previously described [Eq. (3)] weighted-band approach with bands at 540 to 560 nm, 625 to 645 nm, and 820 to 840 nm, which correspond to wavelength regions often used to measure ${\mathrm{StO}}_{2}$ in tissue.^39^^,^^40^ After measuring the optical properties of the oxygenation phantoms, the absorption coefficients at the three bands were used along with Eq. (5) to set up a system of equations to solve for the equivalent melanosome volume fraction, blood volume fraction, and ${\mathrm{StO}}_{2}$, assuming a water volume fraction of 0.5 and fat volume fraction of 0.02.

### 2.8 Statistical Analysis

Spectral similarity is a nontrivial comparison with many different assessment methods existing, each with their own pros and cons.^48^^,^^49^ For our study, two different metrics were selected to compare the target spectra to the measured spectra of the optical property and diffuse reflectance phantoms.

The mean absolute error (MAE) was chosen due to being intuitively interpretable as well as not being overly sensitive to outliers, such as with squared-error metrics, and is not skewed when expected values are near zero, as with percent-error-based metrics. As its name implies, MAE is calculated as the mean of the absolute differences between the measured and expected spectra at each wavelength. For absorption coefficient comparisons, the measured and target spectra were $\log_{10}$-transformed prior to calculating the MAE to bring the values, which will vary over multiple orders of magnitude, onto the same relative scale.

The other chosen metric was the spectral angle mapper (SAM) between the measured and reference spectra, which is a common classification method used in remote sensing and hyperspectral imaging.^48^^,^^49^ SAM represents each spectrum as vectors with dimensionality equal to the number of spectral bands and finds the angle between those two vectors, with smaller angles representing spectra that are more similar in shape to one another and angles closer to 90 deg representing more dissimilar spectra:

$$\mathrm{SAM}(R,S)=\cos^{-1}\frac{R\cdot S}{\left\|R\|\|S\right\|}=\cos^{-1}\;\frac{\sum R_{i}S_{i}}{\sqrt{\sum R_{i}^{2}}\sqrt{\sum S_{i}^{2}}}.$$ (6)

Mathematically, this is determined by taking the inverse cosine of the dot product of the two spectra R and S divided by their root sum of squares. As SAM is an angular measurement, it reflects only on how similar in shape the two spectra are disregarding their relative magnitudes.

The code used to determine the pigment concentrations necessary for the broadband and multiband and diffuse reflectance phantoms have been made available on GitHub along with the normalized pigment properties (https://github.com/AlecWalter/MultipigmentPhantoms).

## 3 Results and Discussion

### 3.1 Pigment Characterization

**Fig. 1:**
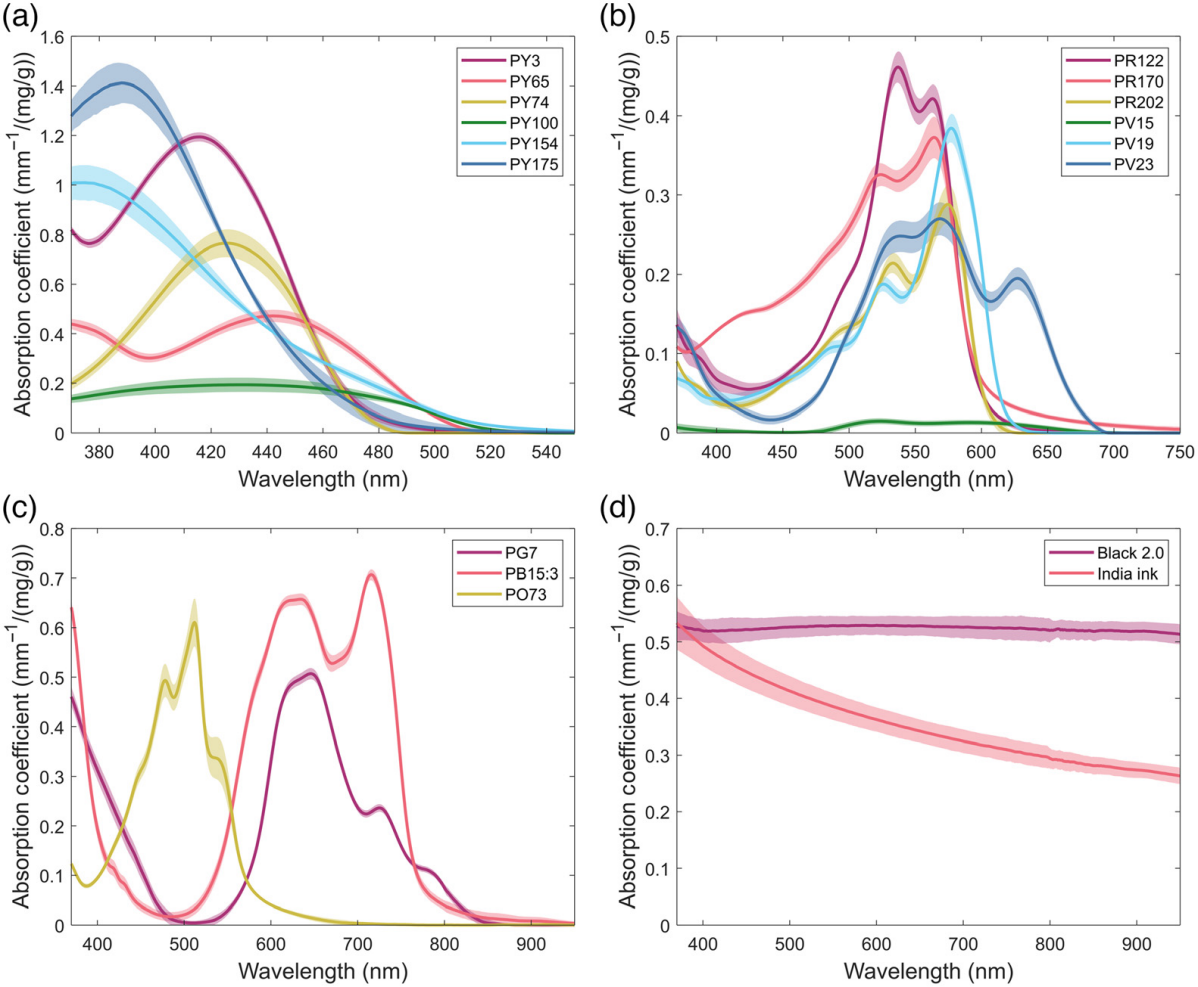
Absorption characterization of colored pigments. Mean measured absorption coefficients of (a) yellow, (b) magenta, (c) miscellaneous, and (d) black pigments normalized to the mass fraction included in epoxy resin. Shaded regions indicate the 95% confidence interval (n=3).

Figure 1 shows the concentration-dependent absorption coefficient spectra of the yellow, magenta, black, and miscellaneous absorbing pigments. As expected, the yellow and magenta pigments were found to have absorption peaks falling between 370 to 480 nm and 500 to 600 nm, respectively. Additionally, it was found that for most of these pigments, the shape of their absorption peaks was similar to what would be expected for tissue in that wavelength region. The yellow pigments mainly have a Gaussian-like shape to their absorption peaks, which are generally similar to the shape of the blue absorption peak of hemoglobin. However, as the Gaussian peaks of the yellow pigments tend to be more broadband as compared to that of hemoglobin, fully matching to that part of the absorption spectrum will be difficult. The magenta pigments, on the other hand, are more closely matched to their tissue counterpart, with most having a bimodal absorption peak similar to that of oxyhemoglobin. Two pigments deviate from this trend, with PV23 having a third absorption peak around 625 nm and PV15 providing a more broadband, but relatively weak, baseline absorption in this region of the spectrum.

Although not being matched to specific tissue spectral features, the three miscellaneous pigments were found to have absorption profiles close to what was desired when they were selected. PO73 was found to have a peak absorption between the yellow and magenta pigments near 500 nm and both PG7 and PB15:3 were found to primarily absorb red wavelengths with significant absorption in the NIR out to 750 nm. For the black pigments, the absorption of India ink was found to be similar to what has been reported previously, decreasing monotonically with wavelength. Interestingly, the absorption of black 2.0 does not follow this same trend, having a nearly flat absorption coefficient between 370 and 950 nm. Although not too major, this difference should allow for better fitting to baseline tissue absorption in the NIR without overly impacting the absorption coefficients in other parts of the spectrum.

**Fig. 2:**
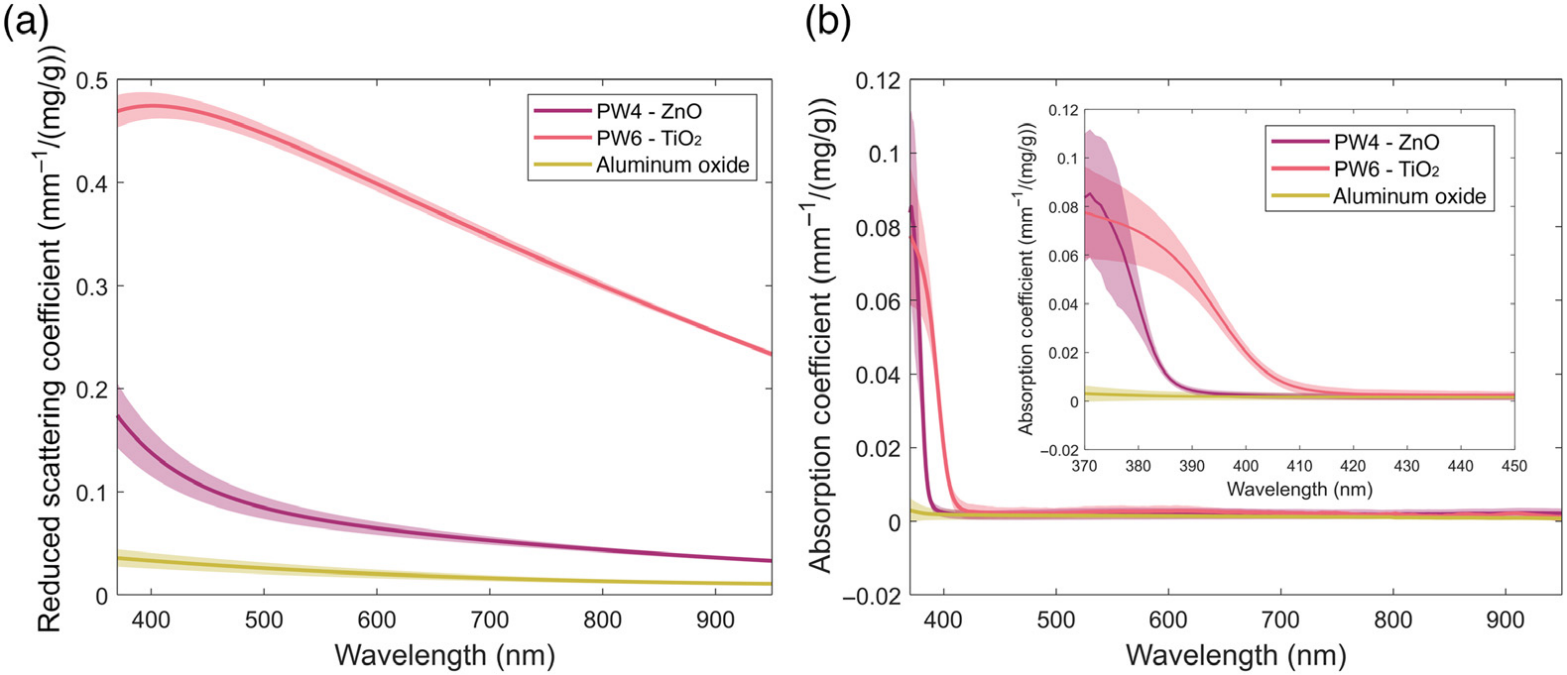
Optical property characterization of white pigments. Mean measured (a) reduced scattering coefficients and (b) absorption coefficients of the white pigments normalized to the mass fraction included in epoxy resin. Shaded regions indicate a 95% confidence interval (n=3). Inset showcases the UV absorption of ZnO and ${\mathrm{TiO}}_{2}$.

Although primarily acting as absorbing agents, the colored pigments were found to contribute measurable amounts of scattering (Fig. S1 in the Supplementary Material). Although the concentration-dependent scattering cannot be neglected, within the range of mass fractions expected to be used the absorbing pigments should only induce small variations in the final reduced scattering coefficients. The majority of the strength and shape of the scattering curve will come from the white pigments, whose concentration-dependent reduced scattering coefficients can be seen in Fig. 2(a).

The relative scattering strength of the three white pigments was, as expected, found to be based on their refractive index mismatch with the epoxy (n=1.56). As seen in Fig. 2(a), PW6 is the most strongly scattering of the white pigments with titanium dioxide having a refractive index of ∼2.6 at 600 nm. This is followed by PW4, with zinc oxide having a refractive index of 2.0 and ${\mathrm{Al}}_{2}O_{3}$ with a refractive index of 1.76. However, as scattering strength can be overcome with increased concentration, the more important difference between the three white pigments is the differences in the shape of their scattering slopes caused by them having varying proportions of Mie and Rayleigh scattering, due to the differences in refractive index and size distribution of the particles. As with the absorbing pigments, these differences should allow for the three while pigments to be combined at different ratios to tweak the shape of the phantom scattering curve to best match that of the target tissue.

Outside of scattering, the absorption coefficients of the white pigments are another major factor of consideration. Although all three were found to have a minimal absorption coefficient throughout the visible and NIR, as seen in Fig. 2(b), both PW4 and PW6 exhibited relatively strong absorption of blue and UV wavelengths, with PW6 absorbing wavelengths shorter than 410 nm and PW4 absorbing wavelengths shorter than 380 nm. Although this may not be relevant for most wavelengths and applications, these absorption peaks limit the ability to use titanium dioxide or zinc oxide as scattering agents when accurate absorption in the blue is required, and especially when high scattering is also required.

Although the pigment characterizations were found to be internally consistent, variations between manufacturers and between batches may result in different concentration-dependent optical properties, as has been shown for India ink.^18^ However, any potential variations that do occur should only be due to amount of pigment originally included in the acrylic paint, as the specified pigments themselves are chemically unique and should thus provide the same absorption peak location and overall shape across batches. Due to this unknown variance, characterizing any materials used prior to use will increase the accuracy of any resulting phantoms and is a generally good practice if possible.

### 3.2 Tissue Optical Property Phantoms

**Fig. 3:**
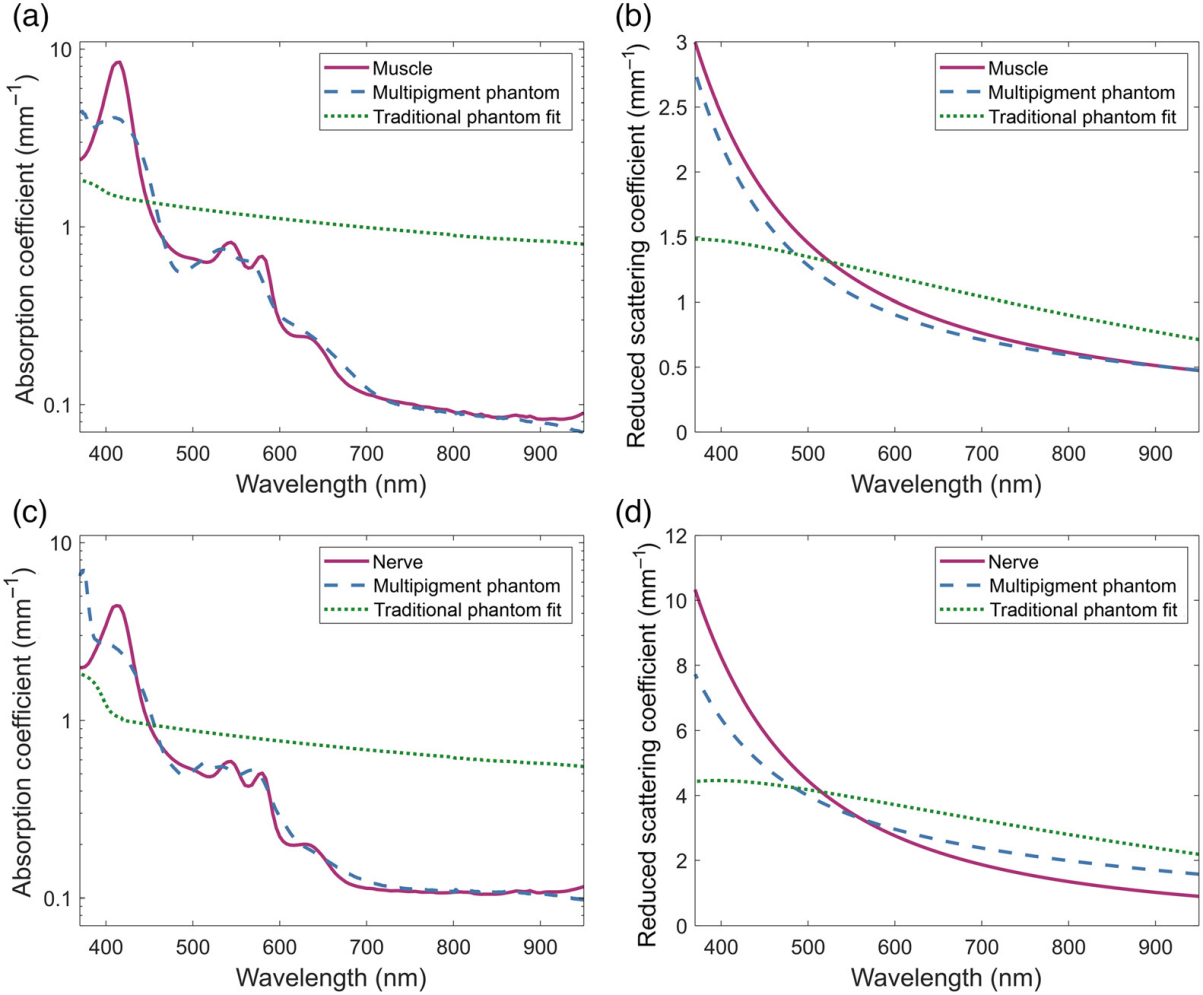
Broadband optical property phantoms. Multipigment phantoms matching the broadband absorption and reduced scattering coefficients of [(a), (b)] muscle and [(c), (d)] nerve tissue from human cadavers.^33^ The spectral optical properties for each were compared to the theoretical best fit of a traditional phantom made using only ${\mathrm{TiO}}_{2}$ and India ink.

Figures 3(a) and 3(b) show the measured absorption and reduced scattering coefficients of the multipigment phantom made to match the optical properties of muscle. Figures 3(c) and 3(d) show the same comparison for the nerve matched phantom. From a visual comparison, it can be seen that the multipigment phantoms are capable approximating the shape of both properties over the entire spectral range of 370 to 950 nm, matching the general scattering curves of both low and high scattering tissue as well as having a nonmonotonic absorption coefficient spectrum that ranges over two to three orders of magnitude.

The quantitative assessment of the similarity of the phantom optical properties to that of their respective tissues can be found in Table 2. From at the analysis of the SAMs, it can be seen that the phantoms are better able to match the shape of the reduced scattering coefficient as compared to absorption coefficient. This is despite the inability of the white pigments to fully match the proportion of Rayleigh scattering present within the tissue scattering spectra. As is apparent from both the SAM and MAE trends, this mismatch results in a more poorly matched scattering spectrum as the average target scattering increases.

**Table 2:** Spectral similarity metrics of the broadband optical property phantoms.

	Muscle		Nerve	
	Multipigment	Traditional fit	Multipigment	Traditional fit
*Absorption*				
MAE	0.055	0.676	0.050	0.535
SAM (°)	22.49	54.27	33.86	46.87
*Scattering*				
MAE	0.092	0.321	0.675	1.447
SAM (°)	1.61	19.47	11.51	27.04

The major reason for the worse overall fit of the absorption coefficient is the discrepancies that occur around the blue peak due to the yellow pigments having a more broadband absorption as compared to the hemoglobin absorption peak. This results in the overall best fit undershooting the maximum absorption value to better match the rest of the values of the slopes of the peak. Additionally, the UV absorption from the white pigments results in further discrepancies in this part of the spectrum, especially for the higher scattering nerve tissue phantom, an issue which was identified during the characterization process.

To contextualize the improvements that the multipigment approach brings to broadband and multispectral phantoms, the muscle and nerve tissue properties were also compared to the theoretical properties that a traditional phantom, consisting only of India ink and titanium dioxide, would have if matched to the full spectrum in the same manner that the multipigment phantoms were. As seen in Fig. 3, the traditional phantom is very limited in the range of values both its absorption and reduced scattering coefficients can take across the spectrum. Although this could be used to roughly approximate the properties of tissue in the NIR, the variable and much greater properties in the visible portion of the spectrum result in an overall poor fit. This limitation is directly reflected in the quantitative similarity metrics of the traditional phantoms. Compared to those of the multipigment phantoms, the theoretical traditional phantoms had MAEs and SAMs that were two to six times greater, representing the high levels of dissimilarity in both spectral shape and in value.

**Fig. 4:**
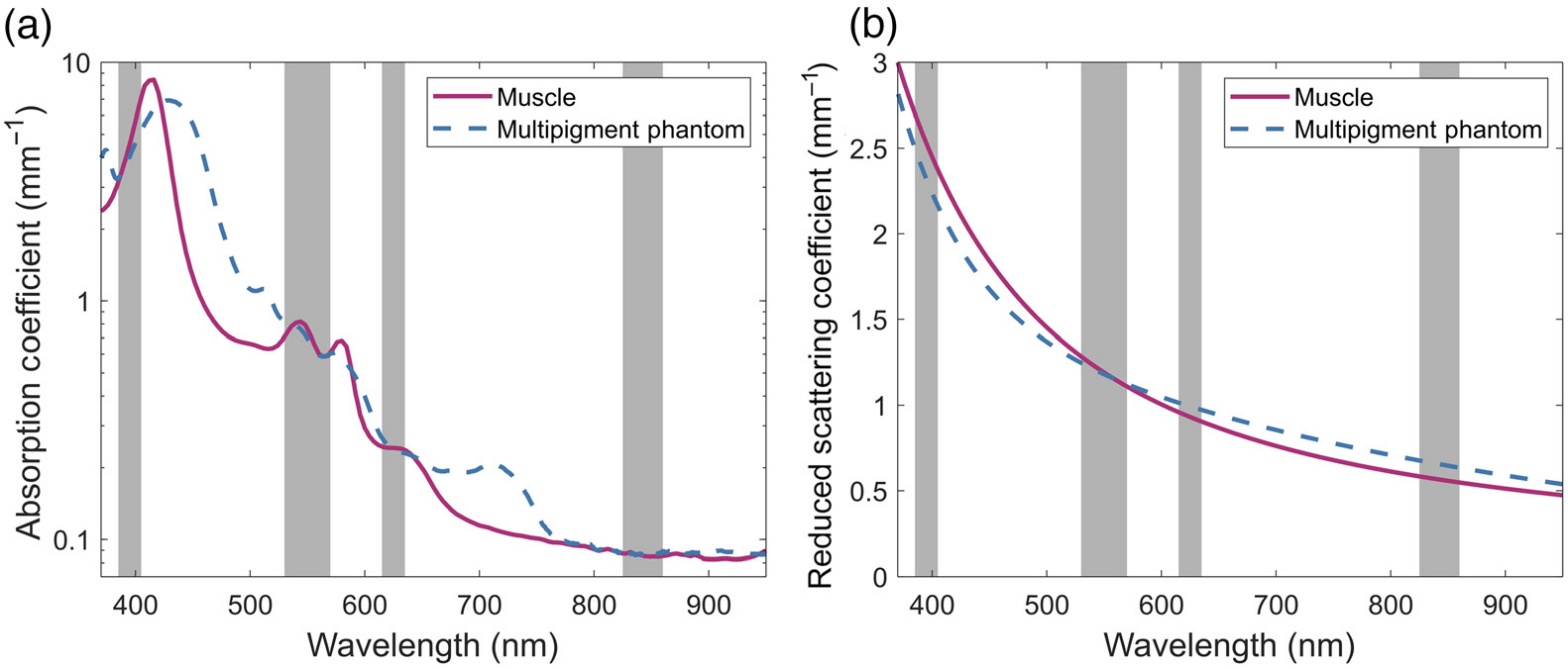
Multiband optical property phantom. Multipigment phantom matching the (a) absorption and (b) reduced scattering coefficients of muscle tissue at four spectral bands in the blue, green, red, and NIR regions of the spectrum, highlighted in gray.

In contrast with the broadband optical property phantoms, Fig. 4 shows the measured properties of the phantom made to match the absorption and reduced scattering coefficients of muscle at four distinct wavelength bands across the blue, green, red, and NIR regions of the spectra. Although the absorption coefficient of this phantom is still broadly shaped like that of tissue, many points outside of the bands deviate away from the target. This mainly occurs where a specific pigment was used to better match the absorption with the band. The two notable examples within this phantom are for the red band, where PB15:3 was used to better match the shape of the shoulder present within the band while also inadvertently introducing an absorption peak around 700 nm, and the blue band, where PY74 was used to try and match the rising slope of the absorption peak despite an overall redshift in the peak location.

When analyzing the quantitative metrics for the band fitted phantom, found in Table 3, the benefits of spectral selection can be seen. For all four bands, the MAE of both the absorption and reduced scattering are very low showcasing the ability of multipigment phantoms to accurately match the optical properties of tissue over multiple orders of magnitude. Additionally, each of these bands were found to have a very small SAM indicating that any wavelength-dependent trends present within the bands can be accurately emulated as well.

**Table 3:** Spectral similarity metrics of the multiband optical property phantom for human muscle tissue.

	Absorption		Reduced scattering	
	MAE	SAM (°)	MAE	SAM (°)
Blue	0.058	5.93	0.209	0.02
Green	0.021	3.66	0.015	0.29
Red	0.013	2.14	0.062	0.71
NIR	0.008	1.03	0.090	0.26
All bands	0.022	6.14	0.080	3.92

Overall, the results from both the broadband and discreet band phantoms showcase that the multipigment approach to phantom design provides more tissue-like optical properties over a broad spectral range. With the ability to independently adjust the optical properties at different regions of the spectrum, multipigment phantoms showcase an increased ability to be tailored toward specific needs and applications.

### 3.3 Diffuse Reflectance Phantoms

**Fig. 5:**
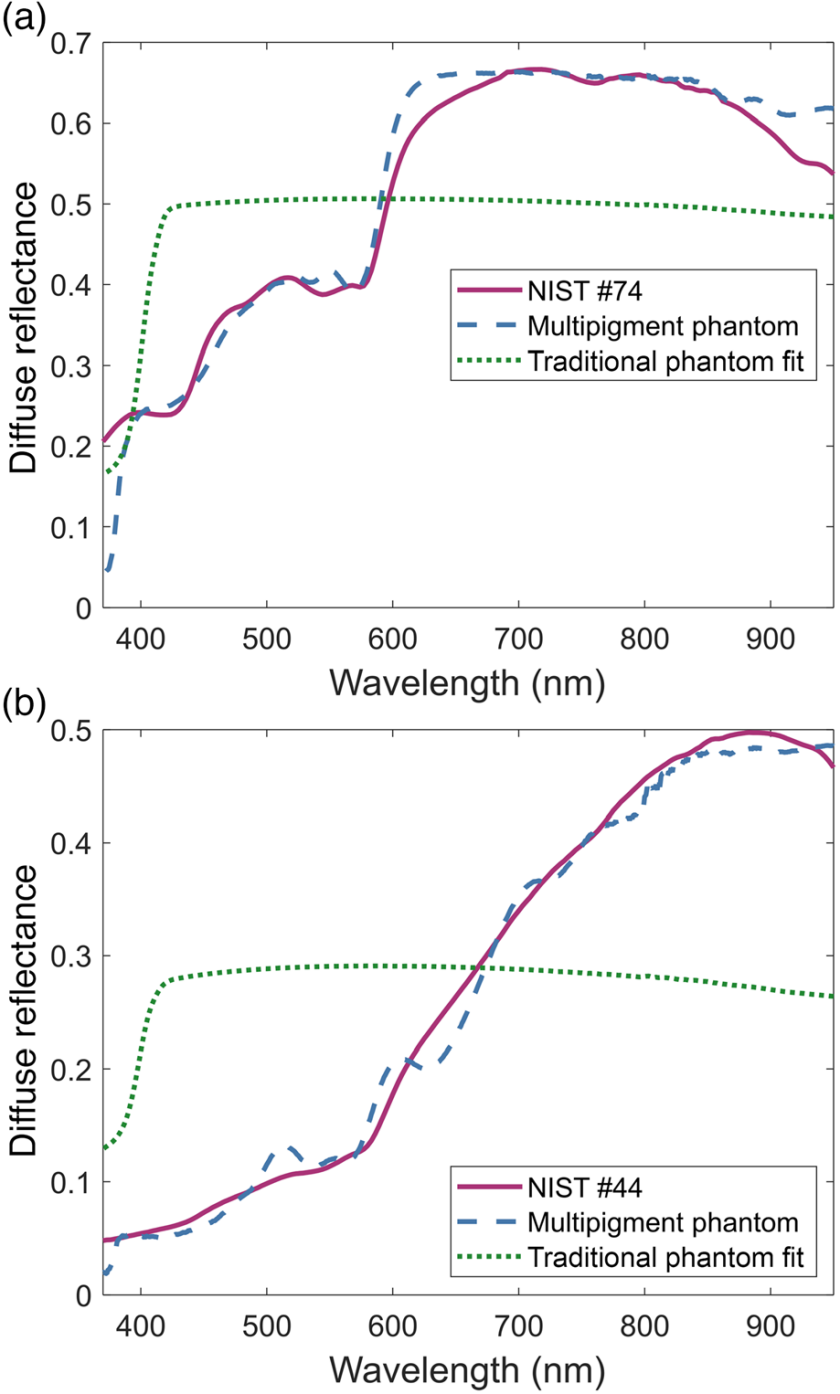
Broadband diffuse reflectance phantoms. Multipigment phantoms matching the broadband diffuse reflectance of (a) pale and (b) melanistic inner forearm skin reported by NIST.^34^ The spectral diffuse reflectances were also compared to the theoretical best fit of a traditional phantom made using only ${\mathrm{TiO}}_{2}$ and India ink.

Figures 5(a) and 5(b) show the measured diffuse reflectance of the reflectance-matched phantoms compared to the NIST reported diffuse reflectance of pale (NIST #74) and melanistic (NIST #44) skin.^34^ From a visual inspection, it can be seen that the multipigment approach, coupled with the wMC model, was able to produce phantoms accurately matching the target diffuse reflectance over most of the spectrum. This is reflected in the quantitative similarity metrics found in Table 4. For both the pale and melanistic skin phantoms, the SAM and the MAE values were close to zero indicating that both the shapes and values of the measured reflectances are close to their targets, despite some apparent deviations.

**Table 4:** Similarity metrics for the multipigment diffuse reflectance phantoms.

	NIST #74		NIST #44	
	Multipigment	Traditional fit	Multipigment	Traditional fit
MAE	0.021	0.126	0.012	0.152
SAM	3.37°	14.09°	2.54°	30.84°

For the pale skin phantom, the major deviations occur at the ends of the spectrum, with the absorption of the scattering agents decreasing the reflectance at the UV end while the lack of variety in absorbers for the NIR prevented accurate matching for that portion of the spectrum. Although the issue in the UV cannot easily be overcome, finding and including new pigments that absorb wavelengths longer than 750 nm would improve the matching in the NIR. In contrast to the pale skin phantom, the main deviations of the melanistic skin phantom occur in the middle of the spectrum. Here the three miscellaneous pigments were unable to bring enough variation outside the yellow and magenta pigments to be combined into an approximation of the smooth absorption profile of melanin, resulting in undesired fluctuations in the measured diffuse reflectance. As such, including more pigments with absorption peaks outside the blue and green regions of the spectrum should result in better emulation of broadband absorbers like melanin.

As with the optical property phantoms, the performance of the multipigment approach when creating broadband diffuse reflectance-matched phantoms was contextualized using the predicted diffuse reflectance of traditional phantoms matched to the full spectrum of the NIST skin reflectances. As seen in Fig. 5, traditional phantoms are expected to have relatively flat diffuse reflectance over the entire spectra, with a sharp decrease in the UV due to the absorption of titanium dioxide. As such, while traditional phantoms could be used to mimic the reflectance of pale skin in the NIR, they are overall limited to matching the value, but not the shape, of the target spectra at a single wavelength.

This limitation is reflected when comparing the quantitative similarity metrics found in Table 4. As compared to the multipigment phantoms, the predicted traditional phantoms were found to have MAEs and SAMs at least 4 times greater, indicating a much greater degree of dissimilarity with the target spectra. This difference is especially apparent for the melanistic skin with the predicted traditional phantom having both an MAE and SAM over 12 times greater than those of the multipigment phantom.

The overall similarity of the phantom and tissue reflectance spectra demonstrates the overall feasibility of using a nonlinear, least squares optimization coupled with a wMC model to produce tissue phantoms based on diffuse reflectance alone. Additionally, these results signify just how poorly traditional phantoms are at mimicking the diffuse reflectance of skin, especially that of darker skin tones. With the multipigment phantom approach’s ability to produce highly accurate, broadband, reflectance-matched phantoms for all skin types, they will be able to better serve as standards when designing and validation optical modalities that minimize racial or ethnic biases due to the presence of melanin.

### 3.4 Oxygenation Saturation Phantoms

**Fig. 6:**
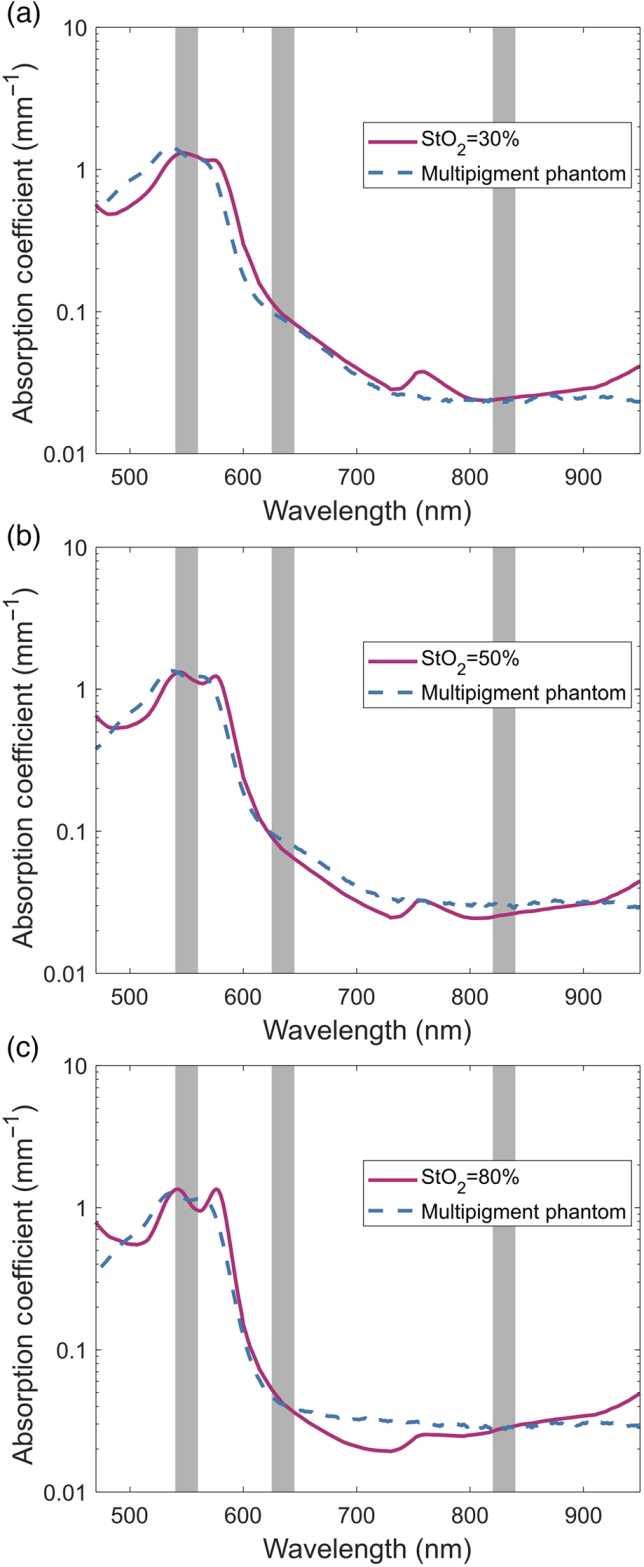
Hemoglobin oxygen saturation phantoms. Multipigment phantoms matching the absorption coefficients of a computational skin model with ${\mathrm{StO}}_{2}$ values of (a) 30%, (b) 50%, and (c) 80% at three wavelength bands centered at 550, 635, and 830 nm, highlighted in gray.

Figures 6(a)–6(c) show the absorption coefficients of the theoretical skin model with blood ${\mathrm{StO}}_{2}$ values of 30%, 50%, and 80%, respectively, compared to the measured absorption coefficients of their corresponding phantoms. The comparison of reduced scattering coefficients can be found in Fig. S2 in the Supplementary Material. From a direct comparison, it can be seen that the phantom absorption coefficients are generally similar to their respective tissue models, with this being especially true within the three selected bands of 540 to 560 nm, 625 to 645 nm, and 820 to 840 nm. Using the absorption coefficients of the phantoms within these three bands, the respective ${\mathrm{StO}}_{2}$, blood volume fraction, and melanosome volume fraction were determined using fixed volume fractions of 0.5 and 0.02 for water and fat. The calculated values were compared to the expected values used to generate the theoretical skin optical properties in Table 5.

**Table 5:** Measured chromophore concentrations of the multipigment oxygen saturation phantoms.

	Expected (%)	Measured (%)	Error (%)
${\mathrm{StO}}_{2}$	30.00	32.94	9.81
Blood volume fraction	4.78	4.62	−3.44
Melanosome volume fraction	0.0255	0.0209	−18.04
${\mathrm{StO}}_{2}$	50.00	49.20	−1.60
Blood volume fraction	4.78	4.58	−4.25
Melanosome volume fraction	0.0255	0.0720	182.35
${\mathrm{StO}}_{2}$	80.00	81.21	1.51
Blood volume fraction	4.78	4.72	−1.29
Melanosome volume fraction	0.0255	0.0277	8.63

Corresponding to the similar optical properties within the three bands, the calculated chromophore values for the three phantoms were found to be physiologically relevant and relatively similar to the expected values from the tissue model. The parameter most prone to error in all three phantoms was found to be the melanosome volume fraction. With the skin model having a minimal amount of melanin, small inaccuracies in the red and NIR absorption bands resulted in small absolute, but large relative, changes in the calculated melanin levels. The worst case of this was for the 50% ${\mathrm{StO}}_{2}$ phantom where the absorption coefficients at 635 and 830 nm being 0.01 and $0.005\;{\mathrm{mm}}^{-1}$ too high, respectively, resulted in a melanosome volume fraction 180% greater than the target value. However, the original volume fraction was set low enough that even this large increase kept the phantom properties within the bounds of pale skin.

Despite this variance in the emulated melanin content of the oxygenation phantoms, the overall blood volume fraction and the ${\mathrm{StO}}_{2}$ were found to be minimally divergent from the target values. The total blood volume fraction was found to be consistent across all three phantoms, tending to be slightly lower than the target value within a 5% margin of error. More importantly, a similar level of accuracy was also observed in the emulated ${\mathrm{StO}}_{2}$ of each phantom, despite the value varying between phantoms, with the 50% and 80% phantoms falling within a 2% error margin and the 30% phantom falling within a 10% error margin. As with the melanosome volume fraction, even with the largest discrepancies the phantoms maintained realistic values that fell within the expected variance of most pulse oximetry systems.^50^ This ability to produce phantoms that can mimic physiologically relevant concentrations of biological chromophores highlights the versatility of the multipigment phantom approach and opens up the possibility to further increase the number of optical modalities that can effectively use tissue phantoms to characterize and validate the performance of their systems.

## 4 Conclusion

In this work, we have outlined a new platform for producing optical phantoms with wavelength-dependent properties that can be adjusted to match those of a desired tissue type over a broad spectral range of 370 to 950 nm through the combination of multiple absorbing and scattering pigments. This opens up the capabilities for solid, shelf-stable phantoms to serve as standards for a wide range of optical techniques which rely on multiple spectral bands or broadband signals. To demonstrate the capability of this approach, we produced multipigment phantoms matched to the optical properties of both human nerve and muscle tissue over the entire wavelength range with greater accuracy as what can be achieved using traditional phantom methods. We also showcased that through the addition of a reflectance model, broadband diffuse reflectance phantoms can be made for both pale and melanistic skin types without knowledge of the underlying tissue optical properties. Finally, we demonstrated that the spectral matching of this approach is accurate enough that inorganic phantoms can be made which emulate physiologically relevant concentrations of tissue chromophores, including representing the same tissue under varying states of hemoglobin ${\mathrm{StO}}_{2}$, a feat that, to our knowledge, had yet to been accomplished. Overall, we believe that this multipigment phantom platform represents a powerful new tool which should increase the availability of solid, stable, and accurate standards for many optical techniques including chromophore imaging, fluorescence imaging, and diffuse reflectance spectroscopy.

## Code, Data, and Materials Availability

The normalized pigment properties and the code used to create the phantom fits have been made available at GitHub: https://github.com/AlecWalter/MultipigmentPhantoms.
