# Supplementary material for: Development of a platform for broadband, spectra-fitted, tissue optical phantoms
Source: J Biomed Opt. 2023 Feb 20;28(2):025001. doi: 10.1117/1.JBO.28.2.025001 (PMC9940728; doi:10.1117/1.JBO.28.2.025001)
Supplement: Supplementary file 1 [file JBO_028_025001_SD001.pdf]

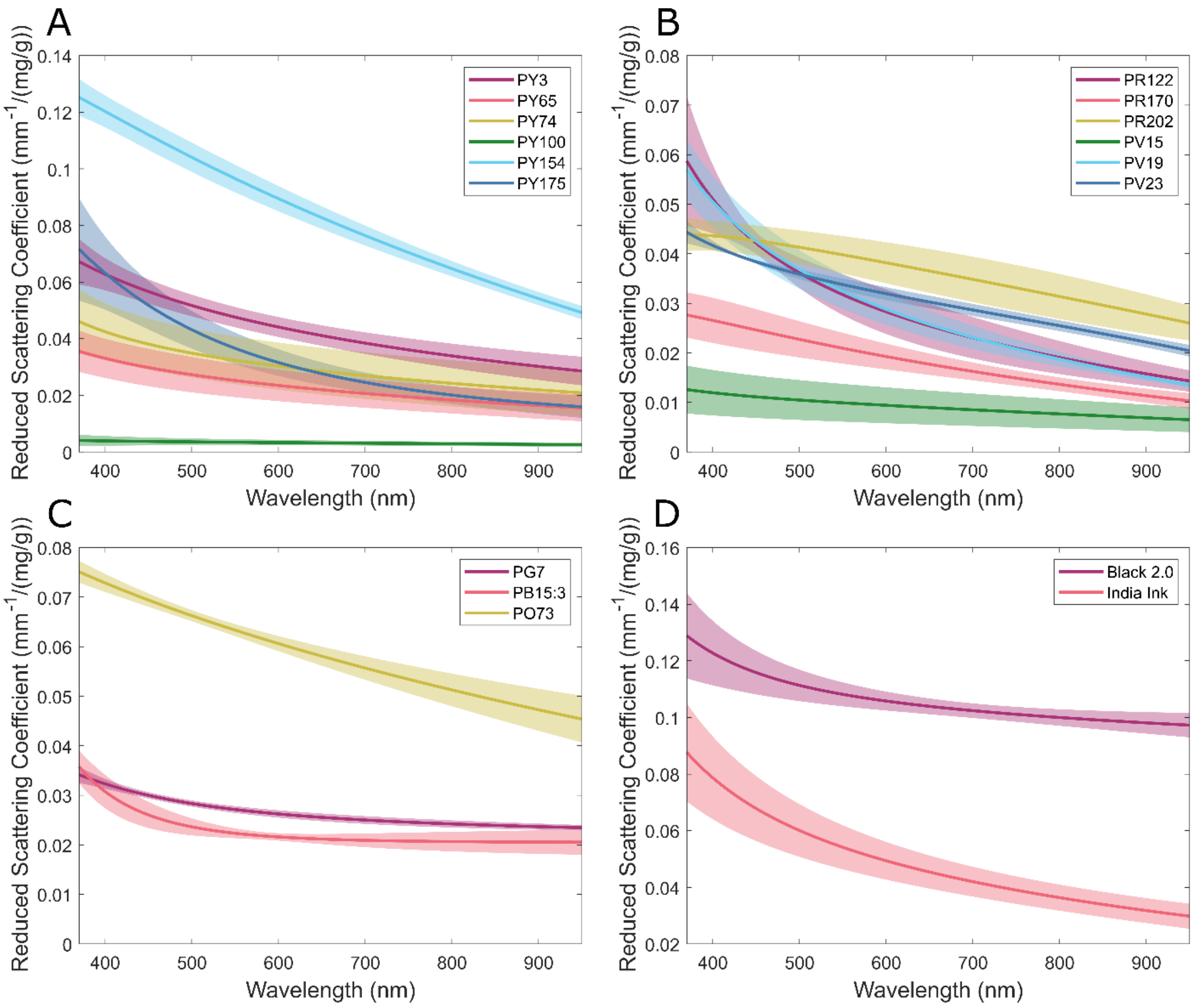

Figure S1. Scattering characterization of colored pigments. Mean measured reduced scattering coefficients of (a) yellow, (b) magenta, (c) miscellaneous, and (d) black pigments normalized to the mass fraction included in epoxy resin. Shaded regions indicate the 95% confidence interval (n=3).

Table S1. Mass fractions of pigments used in the optical property phantoms.

Pigment	Muscle Phantom (mg/g)	Nerve Phantom (mg/g)	Multiband Muscle Phantom (mg/g)
PY3	2.852	1.500	0.000
PY65	0.000	0.000	0.000
PY74	0.000	0.000	8.529
PY100	0.000	0.000	0.000
PY154	0.000	0.000	0.000
PY175	0.746	0.824	0.000
PR122	0.496	0.330	0.000
PR170	0.601	0.000	0.266
PR202	0.163	0.442	0.000
PV15	12.571	4.870	0.000
PV19	0.000	0.000	0.788
PV23	0.000	0.125	0.000
PG7	0.000	0.000	0.000
PB15:3	0.000	0.000	0.136
PO73	0.000	0.203	1.177
Black 2.0	0.131	0.169	0.151
India Ink	0.000	0.000	0.000
PW4	12.287	47.361	11.362
PW6	0.000	0.000	0.000
Al_2O_3	0.000	0.000	0.000

Table S2. Mass fractions of pigments used in the diffuse reflectance phantoms.

Pigment	NIST #74 Phantom (mg/g)	NIST #44 Phantom (mg/g)
PY3	0.367	0.930
PY65	0.134	0.912
PY74	0.000	0.000
PY100	0.346	9.996
PY154	0.000	0.532
PY175	0.228	1.485
PR122	0.194	2.905
PR170	0.000	0.000
PR202	0.199	0.000
PV15	0.000	9.888
PV19	0.157	0.490
PV23	0.000	1.119
PG7	0.000	0.547
PB15:3	0.000	0.000
PO73	0.134	1.205
Black 2.0	0.000	0.000
India Ink	0.039	0.141
PW4	55.904	51.388
PW6	5.028	5.242
Al_2O_3	50.693	49.992

Table S3. Mass fractions of pigments used in the oxygen saturation phantoms.

Pigment	StO_2=30% (mg/g)	StO_2=50% (mg/g)	StO_2=80% (mg/g)
PY3	0.000	0.000	0.000
PY65	0.000	0.000	0.000
PY74	0.000	0.000	0.000
PY100	0.000	0.000	0.000
PY154	0.000	0.000	0.000
PY175	0.000	0.000	0.000
PR122	2.336	2.824	2.693
PR170	0.588	0.000	0.000
PR202	0.000	0.000	0.000
PV15	4.582	3.700	0.000
PV19	0.000	0.000	0.000
PV23	0.000	0.000	0.000
PG7	0.000	0.000	0.000
PB15:3	0.000	0.000	0.000
PO73	0.000	0.000	0.000
Black 2.0	0.025	0.047	0.044
India Ink	0.000	0.000	0.000
PW4	48.377	49.263	49.127
PW6	0.000	0.000	0.000
Al_2O_3	10.480	10.118	9.956

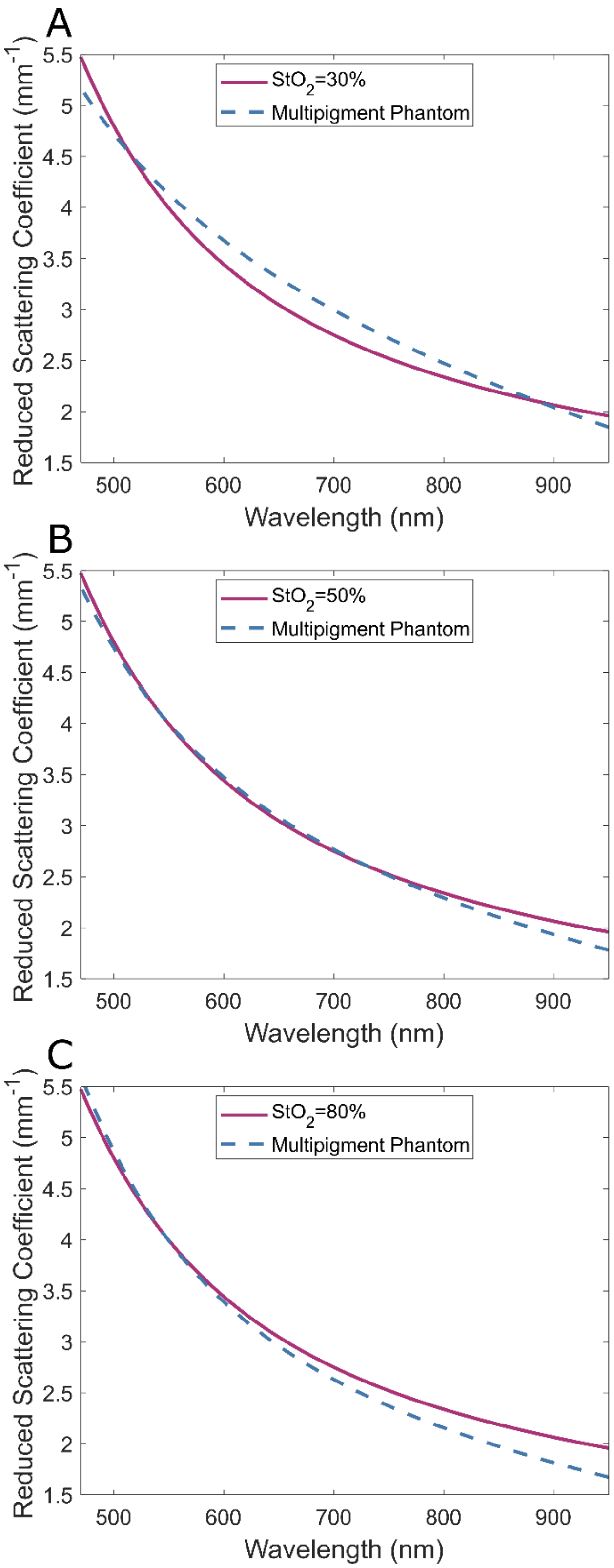

Figure S2. Scattering of hemoglobin oxygen saturation phantoms. Multipigment phantoms matching the reduced scattering coefficients of a computational skin model with oxygen saturation values of (a) 30%, (b) 50%, and (c) 80%.
